# Supplementary material for: Attenuated viral strains of priority pathogens for potential use in controlled human infection model studies: A scoping review
Source: PLoS Negl Trop Dis. 2026 Jan 2;20(1):e0013243. doi: 10.1371/journal.pntd.0013243 (PMC12795465; doi:10.1371/journal.pntd.0013243)
Supplement: S2 File — (DOCX) [file pntd.0013243.s002.docx]

## S2 File. Search strategy

Ovid MEDLINE(R) ALL <1946 to March 27, 2024>

1 Ebola virus.mp. or Ebolavirus/

2 Hemorrhagic Fever, Ebola/ or ebola.mp.

3 1 or 2

4 Lassa virus.mp. or Lassa virus/

5 Lassa fever.mp. or Lassa Fever/

6 4 or 5

7 Nipah virus.mp. or Nipah Virus/

8 Henipavirus Infections/

9 7 or 8

10 Rift Valley Fever/

11 Rift Valley Fever virus.mp. or Rift Valley fever virus/

12 10 or 11

13 Middle East Respiratory Syndrome Coronavirus/ or Middle eastern respiratory.mp.

14 (MERS* or MERS-CoV).mp.

15 13 or 14

16 Chikungunya Fever/ or Chikungunya virus/ or Chikunguya.mp.

17 pseudochallenge*.mp.

18 ((attenuated or mutant or mutated) adj2 (virus* or pathogen* or strain*)).mp.

19 (whole genome adj2 (vaccin* or immun* or challenge*)).mp.

20 (experimental adj3 (vaccin* or immuni* or challenge*)).mp.

21 ((preclinical or pre-clinical) adj2 study).mp.

22 clinical trials as topic/ or clinical trials, phase i as topic/ or clinical trials, phase ii as topic/ or clinical trials, phase iii as topic/ or clinical trials, phase iv as topic/

23 placebo*.mp.

24 controlled trial.mp.

25 CHIM.mp.

26 controlled human infection.mp.

27 human challenge.mp.

28 Vaccines/

29 Vaccination/

30 (((nonhuman or non-human) adj2 primate*) or NHPs).mp.

31 17 or 18 or 19 or 20 or 21 or 22 or 23 or 24 or 25 or 26 or 27 or 28 or 29 or 30

32 3 and 31

33 6 and 31

34 9 and 31

35 12 and 31

36 15 and 31

37 16 and 31

Embase 1947-Present, updated daily

1 Ebola virus.mp. or Ebolavirus/

2 Ebola hemorrhagic fever/ or ebola.mp.

3 1 or 2

4 Lassa virus.mp. or Lassa virus/

5 Lassa fever.mp. or Lassa Fever/

6 4 or 5

7 Nipah virus.mp. or Nipah Virus/

8 Rift Valley Fever/

9 Rift Valley Fever virus.mp. or Rift Valley fever bunyavirus/

10 8 or 9

11 Middle East Respiratory Syndrome/ or Middle eastern respiratory.mp.

12 MERS.mp.

13 11 or 12

14 Chikungunya virus/ or Chikunguya.mp.

15 pseudochallenge*.mp. or virus attenuation/

16 ((attenuated or mutant or mutated) adj2 (virus* or pathogen* or strain*)).mp.

17 (whole genome adj2 (vaccin* or immun* or challenge*)).mp.

18 (experimental adj3 (vaccin* or immuni* or challenge*)).mp.

19 ((preclinical or pre-clinical) adj2 study).mp.

20 clinical study/ or controlled clinical trial/ or multicenter study/ or phase 1 clinical trial/ or phase 2 clinical trial/ or phase 3 clinical trial/

21 placebo*.mp.

22 controlled trial.mp.

23 CHIM.mp.

24 controlled human infection.mp.

25 human challenge.mp.

26 Vaccine/

27 Vaccination.mp.

28 (((nonhuman or non-human) adj2 primate*) or NHPs).mp.

29 15 or 16 or 17 or 18 or 19 or 20 or 21 or 22 or 23 or 24 or 25 or 26 or 27 or 28

30 3 and 29

31 6 and 29

32 10 and 29

33 13 and 29

34 14 and 29

35 7 and 29

Cochrane Central Register of Controlled Trials

Issue 2 of 12, February 2024

#253 Ebola virus or ebola fever

#254 MeSH descriptor: [Hemorrhagic Fever, Ebola] explode all trees

#255 Lassa fever or Lassa virus

#256 MeSH descriptor: [Lassa Fever] explode all trees

#257 Nipah virus

#258 MeSH descriptor: [Henipavirus Infections] explode all trees

#259 Rift valley fever

#260 MeSH descriptor: [Rift Valley Fever] explode all trees

#261 Middle East Respiratory Syndrome

#262 MERS

#263 Chikungunya Fever or Chikungunya virus

#264 vaccin* or immuni* or challenge*

#265 placebo or CHIM

#266 nonhuman primate*

#267 #264 or #265 or #266

#268 #253 or #254

#269 #267 and #268

#270 #255 or #256

#271 #270 and #267

#272 #257 or #258

#273 #272 and #267

#274 #259 or #260

#275 #274 and #267

#276 #261 or #262

#277 #276 and #267

#278 #263 and #267

Clinicaltrials.gov

vaccine | Ebola Virus Disease

Also searched for Ebola, Virus, Viral

vaccine | Rift Valley Fever

vaccine | lassa

vaccine | Nipah Virus Infection

vaccine | MERS (Middle East Respiratory Syndrome)

Also searched for Middle East Respiratory Syndrome

vaccine | Chikungunya Fever

Also searched for Chikungunya

Science Citation Index-Expanded, CABI: CAB Abstracts and Global Health (Web of Science)

|  | Search Query |
| --- | --- |
| #1 | Ebola virus or Ebola fever (Topic) |
| #2 | Lassa virus or Lassa fever (Topic) |
| #3 | TS=(Nipah virus ) |
| #4 | Nipah virus (Topic) |
| #5 | Rift valley fever (Topic) |
| #6 | Middle East Respiratory Syndrome or MERS (Topic) |
| #7 | Chikungunya (Topic) |
| #8 | ((attenuated or mutant or mutated) near/2 (virus* or pathogen* or strain*)) (Topic) |
| #9 | (whole genome near/2 (vaccin* or immun* or challenge*)) (Topic) |
| #10 | (experimental near/2 (vaccin* or immuni* or challenge*)) (Topic) |
| #11 | ((preclinical or pre-clinical) near/2 study) (Topic) |
| #12 | controlled human infection or CHIM or human challenge (Topic) |
| #13 | ((((nonhuman or non-human) near/2 primate*) or NHPs)) (Topic) |
| #14 | #8 OR #9 OR #10 OR #11 OR #12 OR #13 |
| #15 | #14 AND #1 |
| #16 | #14 AND #2 |
| #17 | #14 AND #3 |
| #18 | #14 AND #5 |
| #19 | #14 AND #6 |
| #20 | #14 AND #7 |

Search terms used on PubMed database on 24th February 2025 following conclusion of review to ensure remained up to date (with no additional studies identified):

(Ebola Hemorrhagic Fever or Ebola virus or Lassa virus or Lassa fever or Nipah virus or Rift Valley Fever or Middle eastern respiratory or MERS* or Chikungunya)

AND

(pseudochallenge* or virus attenuation or attenuated virus or mutant virus or vaccin* or pre-clinical study or controlled trial or CHIM)

| **Search** | **Total number of results** | **Number of duplicates deleted** | **Final number of identified studies** |
| --- | --- | --- | --- |
| **EVD** | 6242 | 2083 | 4159 |
| **LV** | 911 | 284 | 627 |
| **NiV** | 620 | 138 | 482 |
| **RVFV** | 1197 | 389 | 808 |
| **MERS-CoV** | 2517 | 362 | 2155 |
| **CHIKV** | 1591 | 397 | 1194 |

Number of studies identified through search strategy. 9425 studies remained after removal of duplication across databases and then 5998 remained after duplication across pathogens and removal of articles identified thorough search terms referencing animal studies.

CHIKV = chikungunya virus, EVD = Ebola virus disease, LV = Lassa virus, MERS-CoV = Middle East respiratory syndrome-related coronavirus, NiV = Nipah virus, RVFV = Rift Valley fever virus
