# Supplementary material for: Attenuated viral strains of priority pathogens for potential use in controlled human infection model studies: A scoping review
Source: PLoS Negl Trop Dis. 2026 Jan 2;20(1):e0013243. doi: 10.1371/journal.pntd.0013243 (PMC12795465; doi:10.1371/journal.pntd.0013243)
Supplement: S3 File — (DOCX) [file pntd.0013243.s003.docx]

## S3 File. Expanded Table of Data Extracted from all included studies

| Author  & Year | Path-ogen | Candi-date name | Study Phase | Institution | Purpose of candidate | Mutation from wild-type | Dosage | Number exposed | Rate of recovery of attenuated virus | Method for detection of virus | Adverse event incidence | Serious adverse events | Mortality | Placebo cohort size/AE/SAE | Follow-up length | Suitable for CHIM | Availability and Regulatory Requirements |
| --- | --- | --- | --- | --- | --- | --- | --- | --- | --- | --- | --- | --- | --- | --- | --- | --- | --- |
| McClain  1998  (1) | CHIKV | TSI-GSD-218 | Phase 1 | USAMRIID | Experimental Live-attenuated vaccine | Two point-mutations on E2 glycoprotein | 4.4 log_10_ PFU  SC | 55 total  N=36 volunteers already immunised against VEEV  N=19 alphavirus-naïve | 36.8% of alphavirus-naïve cohort  (Presumed 7/19) | Amplification in cell culture | 0 AEs in the VEEV-vaccinated group  Overall AE rate not reported  Headache 12%  Fever 6%  Myalgia/  Arthralgia 1%  Local AE 6% | Nil | Nil | 19  Overall AE rate not reported but authors report not significantly different to vaccinated cohort | 12 months | Yes | Sold by ATCC  Unlicenced for human use  Investigatio-nal new drug status closed |
| Edelman  2000  (2) | CHIKV | TSI-GSD-218 | Phase 2 | University of Maryland  USAMRIID  Salk Institute | Experimental Live-attenuated vaccine | Two point-mutations on E2 glycoprotein | 10^5^ PFU  SC | 59 | Not reported | Not reported | Overall AE rates not reported  58% systemic AE  20% local AE  32% related AE | Nil | Nil | 14  64% systemic AE  29% related AE | 12 months | Yes | Sold by ATCC  Unlicenced for human use  Investigational new drug status closed |
| Hoke  2012  (3) | CHIKV | TSI-GSD-218 | Summary of unpublished Phase 1 studies | United States Army | Experimental Live-attenuated vaccine | Two point-mutations on E2 glycoprotein | 3.1 × 10^5^ PFU/mL  0.5 mL IM  2.75x10^4^ PFU/ml  0.5ml SC | 51 total  (Excluding previously published data)  N=30  N=21 | Not reported | Not reported | Overall rates not reported | Not reported explicitly but assumed nil | Not reported explicitly but assumed nil | 31  Overall AE rate not reported but authors report not significantly different to vaccinated cohort | Not reported | Yes | Sold by ATCC  Unlicenced for human use  Investigational new drug status closed |
| Wressnigg  2020  (4) | CHIKV | VLA1553 | Phase 1 | Valneva | Experimental Live-attenuated vaccine | Deletion of part of *nsP3* gene | 3·2 × 10^3^ TCID_50_ /0·1 mL  3·2 × 10^4^  TCID_50_  /mL  3·2 × 10^5^ TCID_50_ /ml  IM | 120 total  N=31  N=30  N=59 | Not reported explicitly but likely very high given inter-quartile ranges of geometric mean titres presented | RT-qPCR | 73.3% total  67.7% low  63.3% med  81.4% high  Related AEs 65.0%  Related severe AEs 10.8% | 0.8%  1 unrelated polytrauma | Not reported explicitly but assumed nil | Nil | 12 months | Yes | Produced by Valneva  Licenced by EMA and MHRA |
| Schneider  2023  (5) | CHIKV | VLA1553 | Phase 3 | Valneva | Experimental Live-attenuated vaccine | Deletion of part of *nsP3* gene | 1 × 10^4^ TCID_50_ per 0·5 mL  IM | 3082  (3093 randomised) | Not reported | Not reported | 62.5% | 1.5%  (46/3082)  2 related SAEs (myalgia and SIADH) | Not reported | 1035  44.8% AE  0.8% SAE | 6 months | Yes | Produced by Valneva  Licenced by EMA and MHRA |
| McMahon  2024  (6) | CHIKV | VLA1553 | Phase 3 | Valneva | Experimental Live-attenuated vaccine | Deletion of part of *nsP3* gene | 1 × 10^4^ TCID_50_ per 0·5 mL  IM | 408 | Not reported | Not reported | Any AE 72.5%  Any related AE 60.5%  Local AE 19.4%  Any related severe AE 2.7% | 1.2%  (5/408)  0 related SAEs | Nil | Nil | 6 months | Yes | Produced by Valneva  Licenced by EMA and MHRA |
| Pittman  2016a  (7) | RVFV | MP-12 | Phase 1 | USAMRIID | Experimental Live-attenuated vaccine | Multiple mutations across all three segments of virus | 10^4.4^ PFU SC  10^4.4^ PFU SC  1:10 dilution  1:100 dilution  1:1000 dilution  10^4.7^ PFU SC  10^3.4^ PFU IM (n=6)  10^4.4^ PFU IM | 69 total  N=4  N=22 across different dilutions  N=10  N=6  N=27 | 16.3%  (7 of 43 assessed) | 1 (2.3%) via direct plaque assay  6 (14.0%) by nucleic acid amplification | Overall AE rates not reported  Of 43 vaccines in fully published data:  Headache 25.6%  Malaise 14.0%  Local AE 9.3% | Nil reported | Nil reported | 13  Overall AE rates not reported but maximum rate of any individual AE = 15.4% | 12 months | Yes | Licence owned by Sabin Vaccine Institute  Unlicenced for human use |
| Pittman  2016b  (8) | RVFV | MP-12 | Phase 2 | USAMRIID | Experimental Live-attenuated vaccine | Multiple mutations across all three segments of virus | 10^5^ PFU IM | 19 | 26.3%  (5/19) | Blind passage of plasma on Vero cells | 89.5%  Headache 57.9%  Malaise 42.1%  Local AE 68.4% | Nil | Nil | Nil | 12 months  (Some for up to 5 years) | Yes | Licence owned by Sabin Vaccine Institute  Unlicenced for human use |
| Leroux-Roels  2024  (9) | RVFV | hRVFV-4s | Phase 1 | Ghent University  Wageningen Bioveterinary Research | Experimental Live-attenuated vaccine | Split M segment genome | 10^4^ TCID_50_  10^5^ TCID_50_  10^6^ TCID_50_ | 60 total  N=20  N=20  N=20 | 0%  (0/60) | RT-qPCR | Overall AE rate not reported  Headache 47%  Fatigue 47%  Local AE 85% | Nil | Nil | 15  Overall AE rate not reported but maximal rate of individual AE = 13% | 6 months | No | Phase 2 study planned |

**Studies included after full-text review**

AE = Adverse Event. ATCC = American Type Culture Collection. CHIKV = chikungunya virus. CHIM = Controlled Human Infection Models. EMA = European Medicines Agency. IM = Intramuscular. MHRA = Medicines and Healthcare products Regulatory Agency. PFU = Plaque forming units. RT-qPCR = quantitative reverse transcription polymerase chain reaction. RVFV = Rift Valley fever virus. SAE = Serious Adverse Event. SC = subcutaneously. SIADH= Syndrome of Inappropriate Antidiuretic Hormone secretion. TCID_50_ = 50% Tissue Culture Infectious Dose. USAMRIID = US Army Medical Research Institute of Infectious Diseases

References for supplementary document only

1. McClain DJ, Pittman PR, Ramsburg HH, Nelson GO, Rossi CA, Mangiafico JA, et al. Immunologic interference from sequential administration of live attenuated alphavirus vaccines. 1998;1(3):634-41.

2. Edelman R, Tacket CO, Wasserman SS, Bodison SA, Perry JG, Mangiafico JA. Phase II safety and immunogenicity study of live chikungunya virus vaccine TSI-GSD-218. 2000;1(6):681-5.

3. Hoke CH, Jr., Pace-Templeton J, Pittman P, Malinoski FJ, Gibbs P, Ulderich T, et al. US Military contributions to the global response to pandemic chikungunya. 2012;1(47):6713-20.

4. Wressnigg N, Hochreiter R, Zoihsl O, Fritzer A, Bezay N, Klingler A, et al. Single-shot live-attenuated chikungunya vaccine in healthy adults: a phase 1, randomised controlled trial. 2020;1(10):1193-203.

5. Schneider M, Narciso-Abraham M, Hadl S, McMahon R, Toepfer S, Fuchs U, et al. Safety and immunogenicity of a single-shot live-attenuated chikungunya vaccine: a double-blind, multicentre, randomised, placebo-controlled, phase 3 trial. 2023;1(10394):2138-47.

6. McMahon R, Fuchs U, Schneider M, Hadl S, Hochreiter R, Bitzer A, et al. A randomized, double-blinded Phase 3 study to demonstrate lot-to-lot consistency and to confirm immunogenicity and safety of the live-attenuated chikungunya virus vaccine candidate VLA1553 in healthy adults. 2024;1(2).

7. Pittman PR, McClain D, Quinn X, Coonan KM, Mangiafico J, Makuch RS, et al. Safety and immunogenicity of a mutagenized, live attenuated Rift Valley fever vaccine, MP-12, in a Phase 1 dose escalation and route comparison study in humans. Vaccine. 2016;34(4):424-9.

8. Pittman PR, Norris SL, Brown ES, Ranadive MV, Schibly BA, Bettinger GE, et al. Rift Valley fever MP-12 vaccine Phase 2 clinical trial: Safety, immunogenicity, and genetic characterization of virus isolates. Vaccine. 2016;34(4):523-30.

9. Leroux-Roels I, Prajeeth CK, Aregay A, Nair N, Rimmelzwaan GF, Osterhaus ADME, et al. Safety and immunogenicity of the live-attenuated hRVFV-4s vaccine against Rift Valley fever in healthy adults: a dose-escalation, placebo-controlled, first-in-human, phase 1 randomised clinical trial. The Lancet Infectious Diseases. 2024;24(11):1245-53.
