# Supplementary material for: Attenuated viral strains of priority pathogens for potential use in controlled human infection model studies: A scoping review
Source: PLoS Negl Trop Dis. 2026 Jan 2;20(1):e0013243. doi: 10.1371/journal.pntd.0013243 (PMC12795465; doi:10.1371/journal.pntd.0013243)
Supplement: S4 File — (DOCX) [file pntd.0013243.s004.docx]

## S4 File. Risk of Bias Assessment


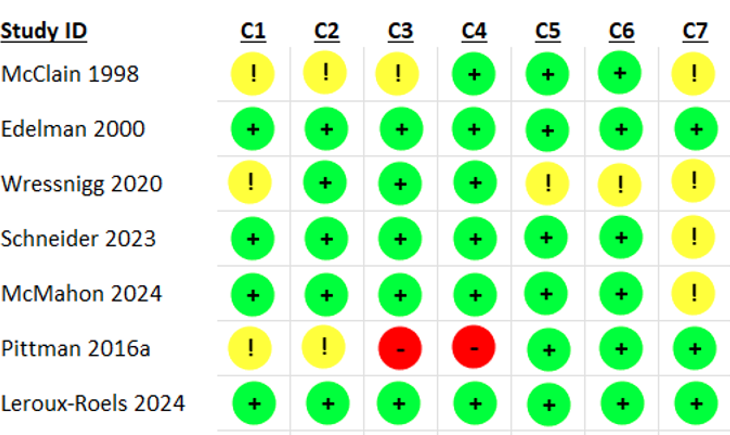


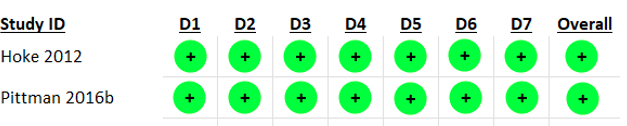


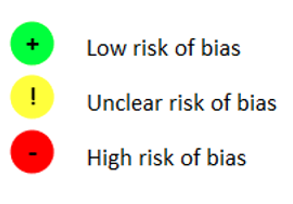


Risk of bias assessment performed on the included studies (Cochrane Collaboration tool for randomised studies (1) and ROBINS-E tool for non-randomised studies (2)).

C1 – random sequence generation (selection bias), C2 – allocation concealment (selection bias), C3 – blinding of participants and personnel (performance bias), C4 – blinding of outcome assessment (detection bias), C5- incomplete outcome data (attrition bias), C6 – selective reporting (reporting bias), C7 – other bias.

D1 – risk of bias due to confounding, D2 – risk of bias arising from measurement of the exposure, D3 – risk of bias in selection of participants into the study, D4 – risk of bias due to post-exposure interventions, D5 – risk of bias due to missing data, D6 – risk of bias arising from measurement of the outcome, D7 – risk of bias in selection of the reported result.

References for supplementary document only

1. Higgins JP, Altman DG, Gøtzsche PC, Jüni P, Moher D, Oxman AD, et al. The Cochrane Collaboration's tool for assessing risk of bias in randomised trials. Bmj. 2011;343:d5928.

2. Higgins JPT, Morgan RL, Rooney AA, Taylor KW, Thayer KA, Silva RA, et al. A tool to assess risk of bias in non-randomized follow-up studies of exposure effects (ROBINS-E). Environ Int. 2024;186:108602.

References for included papers for fig S1:

McClain DJ, Pittman PR, Ramsburg HH, Nelson GO, Rossi CA, Mangiafico JA, et al. Immunologic interference from sequential administration of live attenuated alphavirus vaccines. 1998;1(3):634-41.

Edelman R, Tacket CO, Wasserman SS, Bodison SA, Perry JG, Mangiafico JA. Phase II safety and immunogenicity study of live chikungunya virus vaccine TSI-GSD-218. 2000;1(6):681-5.

Wressnigg N, Hochreiter R, Zoihsl O, Fritzer A, Bezay N, Klingler A, et al. Single-shot live-attenuated chikungunya vaccine in healthy adults: a phase 1, randomised controlled trial. 2020;1(10):1193-203.

Schneider M, Narciso-Abraham M, Hadl S, McMahon R, Toepfer S, Fuchs U, et al. Safety and immunogenicity of a single-shot live-attenuated chikungunya vaccine: a double-blind, multicentre, randomised, placebo-controlled, phase 3 trial. 2023;1(10394):2138-47.

McMahon R, Fuchs U, Schneider M, Hadl S, Hochreiter R, Bitzer A, et al. A randomized, double-blinded Phase 3 study to demonstrate lot-to-lot consistency and to confirm immunogenicity and safety of the live-attenuated chikungunya virus vaccine candidate VLA1553 in healthy adults. 2024;1(2).

Pittman PR, McClain D, Quinn X, Coonan KM, Mangiafico J, Makuch RS, et al. Safety and immunogenicity of a mutagenized, live attenuated Rift Valley fever vaccine, MP-12, in a Phase 1 dose escalation and route comparison study in humans. Vaccine. 2016;34(4):424-9.

Leroux-Roels I, Prajeeth CK, Aregay A, Nair N, Rimmelzwaan GF, Osterhaus ADME, et al. Safety and immunogenicity of the live-attenuated hRVFV-4s vaccine against Rift Valley fever in healthy adults: a dose-escalation, placebo-controlled, first-in-human, phase 1 randomised clinical trial. The Lancet Infectious Diseases. 2024;24(11):1245-53.

Hoke CH, Jr., Pace-Templeton J, Pittman P, Malinoski FJ, Gibbs P, Ulderich T, et al. US Military contributions to the global response to pandemic chikungunya. 2012;1(47):6713-20.

Pittman PR, Norris SL, Brown ES, Ranadive MV, Schibly BA, Bettinger GE, et al. Rift Valley fever MP-12 vaccine Phase 2 clinical trial: Safety, immunogenicity, and genetic characterization of virus isolates. Vaccine. 2016;34(4):523-30.
