# Supplementary material for: Attenuated viral strains of priority pathogens for potential use in controlled human infection model studies: A scoping review
Source: PLoS Negl Trop Dis. 2026 Jan 2;20(1):e0013243. doi: 10.1371/journal.pntd.0013243 (PMC12795465; doi:10.1371/journal.pntd.0013243)
Supplement: Attachment — Submitted filename: 2025_Comments_Hamilton_Plos NTD.docx [file pntd.0013243.s005.docx]

**Comments on the review paper entilted Attenuated viral strains of priority pathogens for potential use in controlled human infection model studies: A scoping review**

**General comments**

This paper is a great review paper dealing with the subject of medical countermeasures (MCM) to control priority viruses considered as high risk for pandemic potential (Ebola, Lassa, Nipah, Rift Valley fever, chikungunya and Middle East respiratory syndrome).

Development of MCMs against these viruses is essential to improve pandemic preparedness. The aim of this scoping review was to systematically examine the literature for viral attenuated strains of CEPI (Coalition for Epidemic Preparedness Innovations) to identify attenuated strains of six CEPI priority pathogens that have been used in humans that could potentially be progressed into novel human challenge models.

Although this systematic review focuses on attenuated viral vaccines, the discussion could have included a table listing other promising vaccines that have already been administered to humans for each of the priority pathogens, in order to provide an overview of vaccines in clinical trials in humans for these different viruses.

**Specific comments**

Figure 1 : Legends : CHIKV = chikungunya should be Chikungunya virus, EBOV, Ebola virus, LV, Lassa virus, MERS should be MERSV Middle East Respiratory Syndrome virus, RVFV = Rift Valley fever virus. MERSV has to be corrected in the figure

Table 2 Legend : same remarks as Figure 1 legends
