# Supplementary material for: Attenuated viral strains of priority pathogens for potential use in controlled human infection model studies: A scoping review
Source: PLoS Negl Trop Dis. 2026 Jan 2;20(1):e0013243. doi: 10.1371/journal.pntd.0013243 (PMC12795465; doi:10.1371/journal.pntd.0013243)
Supplement: Attachment — Submitted filename: CEPI CHIM Response to reviewers comments_Final.docx [file pntd.0013243.s006.docx]

Dear PLOS Neglected Tropical Diseases Editorial team

Thank you for considering our manuscript for your journal and we thank the reviewers for their careful and constructive feedback. Please find a point-by-point discussion of the reviewers’ comments and suggestions. We hope that with the edits outlined below, you would accept our manuscript for publication in your journal.

Many thanks

Dr Oliver Hamilton and Dr Ben Morton, on behalf of all authors

**Methods**

*Reviewer #1: Standard structured literature review, hampered by only partial access to old publications where results were not appropriately published.*

*Reviewer #2: Objectives of the study are clearly articulated. As it was a literature review, there was no testable hypothesis and no study population. Sample size and statistical analyses were not applicable. There are no concerns about ethical or regulatory requirements. The study design is appropriate for the stated objective of systematically reviewing literature.*

*Reviewer #3: The objectives of the study care articulated with a clear testable hypothesis stated in this review paper. The Mat and methods are clearly described and appropriate for the hypothesis being tested.*

**Response:**

Thank you to the reviewers for this positive feedback.

**Results**

*Reviewer #1: As a literature review the manuscript is sufficient. A table for each of the pathogens studied with key study data would be useful, certainly more so than Figure 2 which could go to supplement. Some more space could go to the AE description*

**Response:**
Thank you – we have moved the Risk of Bias Assessment (formally Figure 2) to the supplementary appendix (Figure S1) as suggested. Please note, supplementary table S2 expands the key study data such as dosage, placebo cohort details and the AE description. We have now signposted this more clearly by providing more information in the legend for “Table 1 describes the characteristics of included studies and each pathogen is described separately, with further detail in Supplementary Table S2”.

*Reviewer #2: The analysis is aligned with the analysis plan and the results are clearly and completely presented. Figures are of sufficient quality for clarity.*

*Reviewer #3: the analysis presented matches the analysis plan. The results are clearly and completely presented. The legends of the figure have to be corrected*

**Response:**

Thank you – the legends of the figure have been corrected (see “Editorial and Data Presentation Modifications?” below)

**Conclusions**

*Reviewer #1: I am left underwhelmed by the lack of thoughtful and more extensive consideration in the discussion of what uses should/could an attenuated virus be put to, how to engineer it, manufacture, test in a pre-clinical fashion. Now with sophisticated genetic tools and cell culture it would seem to be important to highlight the potential of modern approaches to this important issue. Deficiencies in the reporting of older studies by the US military should be pointed out.*

**Response:**

Whilst we agree with the reviewer that exploration of how best to produce attenuated viruses would be a very interesting topic, the object of our review was to identify existing attenuated viruses that have been subject to early phase human trials. The overarching aim of this work was to identify “off the shelf” viruses suitable for use in CHIM research. We recognise this as a limitation and have highlighted that creation of new attenuated mutant viruses should be explored further in our conclusion. Furthermore, we have added the word “existing” in a number of places in the discussion to help highlight the aim of our work. Further, we now also explicitly discussed the limitations of US military reporting in our discussion and thank the reviewer for this suggestion.

*Reviewer #2: The conclusions are supported by the data presented. There was no description of limitations of the analysis. The authors do discuss how these data could be helpful in the development of controlled human infection models that may accelerate future development of vaccines against CEPI Priority Pathogens, which would be highly relevant to public health.*

**Response:**

Thank you for highlighting this issue. We have now more clearly signposted our limitations section in the discussion and expanded this to include issues around incomplete reporting from the US military publications.

*Reviewer #3: Conclusions are supported by the data/figures/tables presented*

**Editorial and Data Presentation Modifications**

*Reviewer #1: (No Response)*

*Reviewer #2: Please consider the following minor revisions:
Lines 49-50: "...VLA1553 for chikungunya is in the most advanced stage of development..." I consider this to be an understatement because VLA1553/Ixchiq is licensed in the European Union, therefore I would consider it's clinical development to be essentially complete, rather than at an "advanced stage". Consider instead, "...VLA1553 is a licensed and commercially available vaccine product suitable for use in a controlled human infection model.*

**Response:**

Thank you. We have amended the wording as suggested.

*Line 293: Consider clarifying here that Ixchiq is the commercial name for VLA1553. As currently written, it isn't completely clear these are the commercial and development code names for the same vaccine.*

**Response:**

Thank you. We have corrected this.

*Lines 301-302: As was done later for some of the other attenuated viruses, consider adding a comment about the risk of reversion of VLA1553 to a more virulent strain based on the nsP3 deletion. It isn't obvious why this deletion would "eliminate" the risk of reversion.*

**Response:**

Thank you for raising this point. We have changed the word “eliminate” to “minimise” and added a further sentence in the discussion addressing fears over reversion: “The use of attenuated virus raises concerns about reversion to wild-type, however to date there has been no reports of this for VLA1553 and the deletion of a large part of the gene nsP3 renders this unlikely”.

*Line 308: Please clarify if subjects in this clinical study received 3 different dose levels (e.g. low, medium, and high doses) or if each subject received 3 immunizations (and if so, at what interval?).*

**Response:**

We apologise for the confusion and have clarified that participants received a single-shot and that doses were escalated.

*Line 312: I apologize for being somewhat pedantic, but viremia refers to detection of replication competent viruses in blood, for example by plaque assay. qRT-PCR only detects viral RNA, not the presence of replication-competent viruses. Consider rewording: "...a transient increase in viral RNA after the first dose was detected by qRT-PCR..."*

**Response:**

We thank the reviewer for this observation and have made the suggested change and also changed other similar incorrect references to ‘viraemia’ throughout the manuscript.

*Line 315: "Shedding of attenuated viruses was only detected at one time point..." Given that CHIKV is transmitted by an insect vector, it isn't clear what "shedding" refers to. Does this mean attenuated viruses were detected in serum? Please clarify.*

**Response:**

Again, we apologise for the confusion and have clarified this as “urinary” shedding, which is how this issue was described in the published study.

*Line 325: Consider adding details on the SAEs deemed related. Given that this virus is being proposed for use in a CHIM, safety is a paramount concern.*

**Response:**

We agree with the reviewer’s concern and have added the following detail on the SAE: “(one mild myalgia in a 58-year-old patient with known fibromyalgia leading to a five-day hospitalisation for investigation and one admission for presumed syndrome of inappropriate antidiuretic hormone secretion and atrial fibrillation in a 66-year-old patient following a fever on day 11)”.

*Line 326: "Viraemia was not reported in this study" Please clarify if this means the authors of the study did not report on this outcome, or if viremia was assayed for, but not observed.*

**Response:**

Thank you – we have clarified that for both this and the subsequent study that no attempt was made to recover the attenuated virus.

*Line 329: VLA1553, not "VLA1153".*

**Response:**

Thank you for observing this typographical error, which we have corrected.

*Lines 329-330: the license of Ixchiq has been revoked by the US FDA, therefore this section needs to be updated to reflect the current state.*

**Response:**

Thank you for highlighting this important issue – we have updated this section with the relevant safety events (with references) since initial submission, which now reads “However, use has recently been paused in adults aged over 65 in Canada and the UK due to concern regarding post-marketing safety reports, including three deaths, in this subpopulation (84-86). Having initially licenced Ixchiq for use in the USA, the Food and Drug Administration has since suspended this licence for all adults due to these safety concerns (84).”

*Lines 339-340: Consider adding a comment about risk of TSI-GSD-218 reverting to virulence, given that attenuation is mediated by only two point mutations. Given that this strain is being proposed for use in CHIMs, stability of the attenuation is a prime concern.*

**Response:**

Thank you for raising this important concern– we have added this to the discussion and also added that the attenuation is “only” mediated by a two-point mutation.

*Line 350: "...randomised 4:1 to TSI-GSD-218..." I assume this means 4 vaccine to 1 placebo, but please clarify.*

**Response:**

We have clarified this to “73 healthy volunteers were recruited to a randomised, placebo-controlled trial of vaccination with TSI-GSD-218 (4:1 vaccine to placebo)”

*Lines 408 and 542: The reported attack rate of 26.3% for MP-12 is relatively low compared to strains used for other CHIMs and would have significant implications on the number of subjects needed to enroll in order to sufficiently power a CHIM study testing a vaccine candidate. Consider adding comment on this in the Discussion of the suitability of MP-12 for use in a CHIM.*

**Response:**

This is a highly relevant point that has now been added to the discussion, which now reads: “The recovery rate of MP-12 in participants is low at 16.3-26.3%, which would necessitate a large sample size in a hypothetical CHIM (45, 47). However, these rates are based on techniques of direct plaque assay, nucleic acid amplification or blind passage through Vero cells and more work would be required to determine the attack rate using contemporary diagnostic assay techniques. In order to progress MP-12 into a CHIM, a method of recovering the strain by RT-qPCR would need to be developed.”

*Lines 439-440. The statement that there are no licensed antiviral medications for EBOV is incorrect. There are two monoclonal antibody therapies licensed by the US FDA for EBOV: Inmazeb and Ebanga. These should be mentioned here. The reference cited for this statement (#117 Jacob et al. Nat Rev Dis Primers) was published in February 2020, whereas the two mAb therapies were approved in October and December 2020, respectively. Consider citing a more recent reference that describes Inmazeb and Ebanga.*

**Response:**

We thank the reviewer for highlighting this oversight. We have now corrected it to include these two treatments and cited a more recent reference as suggested.

*Reviewer #3: Specific comments
Figure 1 : Legends : CHIKV = chikungunya should be Chikungunya virus, EBOV, Ebola virus, LV, Lassa virus, MERS should be MERSV Middle East Respiratory Syndrome virus, RVFV = Rift Valley fever virus. MERSV has to be corrected in the figure
Table 2 Legend : same remarks as Figure 1 legends*

**Response:**

Thank you for the comments on our legends. We have been through the manuscript and all figures/tables to ensure there is a consistent use of acronyms for both the viruses and the resultant clinical diseases. We have amended the legends for both figures so that they now read “CHIKV = chikungunya virus” (however we do not believe it should be capitalised according to convention), “EVD = Ebola virus disease” (as EBOV is typically specific for Zaire ebolavirus), “LV = Lassa virus” (as suggested), “RVFV = Rift valley fever virus” (as suggested), “MERS-CoV = Middle East respiratory syndrome-related coronavirus” (as per convention) and “NiV = Nipah virus” (as per convention).

**Summary and General Comments**

*Reviewer #1: (No Response)*

*Reviewer #2: The authors have conducted a systematic literature review to identify potential attenuated virus strains of several CEPI Priority Pathogens that could be used in controlled human infection model (CHIM) studies. This comprehensive review does an excellent job of summarizing the various studies conducted with these virus strains. Hopefully this review will stimulate the development of these and other attenuated strains for use in future CHIM studies.*
*General comment: the description of the strains focused on their replication in clinical studies, which is appropriate as the primary consideration for a CHIM strain. However, please consider adding comments about the immunological responses elicited by these strains, if known, as this could be compared with the immunological responses to virulent wild-type strains and thus serve as an important indicator of how useful and valid the attenuated strains would be as challenge strains.*

**Response:**

We are grateful for the reviewer’s enthusiastic response to our work and thank them for this suggestion regarding immunological responses. We have mentioned the immunological response to VLA1153 is similar to natural infection the discussion with an additional reference: Kosulin K, Brasel TL, Smith J, Torres M, Bitzer A, Dubischar K, et al. Cross-neutralizing activity of the chikungunya vaccine VLA1553 against three prevalent chikungunya lineages. Emerg Microbes Infect. 2025:2469653.

The authors of the Phase 1 study of VLA1553 report “that maximal peak titres of antibodies post exposure to VLA1553 were similar to sera analysed from patients after natural CHIKV infection” – however, this data is unpublished and journal regulations regarding such data prohibit us from including this.

There is limited available literature on the immunological response of older strains compared to wild-type.

*Reviewer #3: Although this systematic review focuses on attenuated viral vaccines, the discussion could have included a table listing other promising vaccines that have already been administered to humans for each of the priority pathogens, in order to provide an overview of vaccines in clinical trials in humans for these different viruses.*

**Response:**

Whilst we agree with the reviewer that a comprehensive review of all promising vaccines for priority pathogens is an interesting avenue for exploration, it deserves its own scoping review and would need a thorough search to ensure all candidates were included. This is outside of the scope of identifying existing attenuated viruses that have been subject to early phase human trials, however we have added the G-finder report (reference 153) to signpost the interested reader.

*Specific comments
Figure 1 : Legends : CHIKV = chikungunya should be Chikungunya virus, EBOV, Ebola virus, LV, Lassa virus, MERS should be MERSV Middle East Respiratory Syndrome virus, RVFV = Rift Valley fever virus. MERSV has to be corrected in the figure
Table 2 Legend : same remarks as Figure 1 legends*

**Response**

We have addressed this in the above section “Editorial and Data Presentation Modifications?”

**Comments on the review paper entilted Attenuated viral strains of priority pathogens for potential use in controlled human infection model studies: A scoping review**

***General comments***

*This paper is a great review paper dealing with the subject of medical countermeasures (MCM) to control priority viruses considered as high risk for pandemic potential (Ebola, Lassa, Nipah, Rift Valley fever, chikungunya and Middle East respiratory syndrome).*

*Development of MCMs against these viruses is essential to improve pandemic preparedness. The aim of this scoping review was to systematically examine the literature for viral attenuated strains of CEPI (Coalition for Epidemic Preparedness Innovations) to identify attenuated strains of six CEPI priority pathogens that have been used in humans that could potentially be progressed into novel human challenge models.*

*Although this systematic review focuses on attenuated viral vaccines, the discussion could have included a table listing other promising vaccines that have already been administered to humans for each of the priority pathogens, in order to provide an overview of vaccines in clinical trials in humans for these different viruses.*

**Response:**

We thank the reviewer for their positive feedback. We have answered the point about listing other promising vaccines above, which we believe warrants a scoping review in itself and is outside of the aims of this work.

***Specific comments***

*Figure 1 : Legends : CHIKV = chikungunya should be Chikungunya virus, EBOV, Ebola virus, LV, Lassa virus, MERS should be MERSV Middle East Respiratory Syndrome virus, RVFV = Rift Valley fever virus. MERSV has to be corrected in the figure*

*Table 2 Legend : same remarks as Figure 1 legends*

**Response:**

As above, we have been through the manuscript and all figures to ensure there is a consistent use of acronyms for both the viruses and the resultant clinical diseases. We have amended the legends for both figures so that they now read “CHIKV = chikungunya virus” (we do not believe it should be capitalised according to convention), “EVD = Ebola virus disease” (as EBOV is specific for Zaire ebolavirus), “LV = Lassa virus” (as suggested), “RVFV = Rift valley fever virus” (as suggested), “MERS-CoV = Middle East respiratory syndrome-related coronavirus” (as per convention) and “NiV = Nipah virus” (as per convention).
